# Dietary guidance for lutein: consideration for intake recommendations is scientifically supported

**DOI:** 10.1007/s00394-017-1580-2

**Published:** 2017-11-17

**Authors:** Katherine M. Ranard, Sookyoung Jeon, Emily S. Mohn, James C. Griffiths, Elizabeth J. Johnson, John W. Erdman

**Affiliations:** 1 Division of Nutritional Sciences, University of Illinois at Urbana-Champaign, Urbana, IL USA; 2 Jean Mayer US Department of Agriculture Human Nutrition Research Center on Aging, Tufts University, Boston, MA USA; 3 Science and International Affairs, Council for Responsible Nutrition-International, Washington, DC, USA; 4 Department of Food Science and Human Nutrition, University of Illinois at Urbana-Champaign, 455 Bevier Hall, 905 S. Goodwin Ave, Urbana, IL 61801 USA

**Keywords:** Lutein, Intake recommendations, Bioactives, Visual performance, Macular degeneration

## Abstract

Lutein, a yellow xanthophyll carotenoid found in egg yolks and many colorful fruits and vegetables, has gained public health interest for its putative role in visual performance and reducing the risk of age-related macular degeneration. The National Academies of Sciences, Engineering and Medicine’s recommended Dietary Reference Intakes (DRIs) focus on preventing deficiency and toxicity, but there is a budding interest in establishing DRI-like guidelines for non-essential bioactives, like lutein, that promote optimal health and/or prevent chronic diseases. Lupton et al. developed a set of nine criteria to determine whether a bioactive is ready to be considered for DRI-like recommendations. These criteria include: (1) an accepted definition; (2) a reliable analysis method; (3) a food database with known amounts of the bioactive; (4) cohort studies; (5) clinical trials on metabolic processes; (6) clinical trials for dose–response and efficacy; (7) safety data; (8) systematic reviews and/or meta-analyses; (9) a plausible biological rationale. Based on a review of the literature supporting these criteria, lutein is ready to be considered for intake recommendations. Establishing dietary guidance for lutein would encourage the consumption of lutein-containing foods and raise public awareness about its potential health benefits.

## Introduction

Lutein is a yellow xanthophyll carotenoid found in egg yolks and many fruits and vegetables, most notably leafy green vegetables like kale and spinach [1]. Lutein and its isomers, zeaxanthin and *meso*-zeaxanthin, are the only carotenoids that accumulate in the fovea of the human retina and constitute macular pigment (MP) [2]. The biological specificity for these carotenoids suggests that they have a role in the visual system. Increased dietary lutein/zeaxanthin intake raises serum concentrations and MP density. These markers are associated with improvements in visual function and a reduced risk of age-related macular degeneration (AMD) [3]. However, the average American adult consumes only ~ 1–2 mg lutein/day [4], which may not be enough to attain these health benefits.

There is growing enthusiasm for setting intake recommendations for non-essential dietary bioactives, such as lutein, that promote optimal health and/or prevent chronic diseases [5–7]. This is an extension of the traditional approach that is currently used for setting dietary reference intake (DRI) values, which focuses on preventing deficiency and toxicity of essential nutrients, e.g., vitamins and minerals. Establishing intake guidelines for lutein could encourage the consumption of lutein-containing foods and subsequently improve visual health.

Lupton et al. [8] developed a set of nine criteria to determine whether a bioactive has sufficient evidence to be considered for DRI-like recommendations. These criteria include: (1) an accepted definition; (2) a reliable analysis method; (3) a food database with known amounts of the bioactive; (4) cohort studies; (5) clinical trials on metabolic processes; (6) clinical trials for dose–response and efficacy; (7) safety data; (8) systematic reviews and/or meta-analyses; (9) a plausible biological rationale.

Using the existing literature, we assessed how well lutein meets these nine criteria. This topic was briefly discussed by Johnson in 2014, but in this article, we elaborate on each criterion and include more recent evidence [3, 9]. It was not always possible to distinguish between lutein and its isomer zeaxanthin in our discussion, as many studies and dietary databases report a combined ‘lutein + zeaxanthin’ value. Lutein and zeaxanthin are both concentrated in the macula, are present in many of the same foods, and offer similar potential health benefits. Because of the close relationship between these isomers, we assert that a given criterion is fulfilled if the evidence supports either lutein alone or a combination of lutein/zeaxanthin.

### Commonly accepted definition

#### Criterion rationale

With a commonly accepted definition, a database of efficacy can be created for the bioactive.

#### How lutein meets this criterion

Lutein is a yellow xanthophyll carotenoid with a singular molecular formula (C_40_H_56_O_2_), an International Union of Pure and Applied Chemistry (IUPAC) name [(3R,3′R)-*β,β*-carotene-3,3′-diol], and a chemical abstracts services (CAS) registry number (127-40-2).

### Analysis method consistent with definition

#### Criterion rationale

An established analysis method allows for comparison across laboratories and studies. Intake values can be measured to determine whether populations are meeting dietary recommendations.

#### How lutein meets this criterion

Lutein content in foods or tissues is commonly analyzed and quantified via high-performance liquid chromatography (HPLC). The National Institute of Standards and Technology (NIST) developed standard reference material (SRM) 968e, which includes lutein among other carotenoids, fat-soluble vitamins, and cholesterol in human serum [10]. SRM 968e can be used to validate methods for determining total lutein concentrations and/or quality assurance [10]. Several commonly used extraction and HPLC analysis methods include Epler et al. [11] and Sander et al. [12], which employ C18 and C30 analytical columns, respectively. The Yeum et al. [13] HPLC method (C30 analytical column) is also frequently used. The US Pharmacopeia (USP) has three quality monographs for (1) Lutein, (2) Lutein preparation, and (3) Lutein capsules. The USP Lutein Reference Standard [14] is used for testing lutein content. USP test methods use L3 porous silica columns that separate lutein and zeaxanthin (USP-NF 40-35) [15]. Some HPLC methods cannot separate lutein and its isomer zeaxanthin; this is one reason why ‘lutein + zeaxanthin’ are often reported together.

### Food databases

#### Criterion rationale

Food databases indicate the quantity of the bioactive in the food supply. This information can be used to determine intake values.

#### How Lutein meets this criterion

Green leafy vegetables, egg yolks, and some orange/yellow fruits and vegetables are rich in lutein and zeaxanthin. The United States Department of Agriculture (USDA) Food Composition Database reports ‘lutein + zeaxanthin’ content in foods [1]. Others have analyzed foods such as fruits, vegetables, corn, and egg products specifically for lutein content [16].

### Prospective cohort studies

#### Criterion rationale

The level of efficacy for a bioactive can be set based on dose–response data.

#### How lutein meets this criterion

At the highest percentile groupings of intake (~ 3–5 mg/day), lutein/zeaxanthin reduced the risk of early [17], intermediate [18], and advanced [19] AMD. Other cohort studies reviewed by Sabour-Pickett et al. show an inverse relationship between dietary or serum lutein/zeaxanthin and AMD [20].

### Clinical trials on digestion, absorption, activation, transport, and excretion

#### Criterion rationale

Factors that impact metabolism of the bioactive can be determined to inform intake recommendations.

#### How lutein meets this criterion

Plasma kinetics studies have been conducted to show the absorption and excretion of lutein following a regimen of daily lutein supplements [21]. Similar to other carotenoids, the bioavailability of lutein increases when co-consumed with fat [22]. The food source and matrix also influence bioavailability: lutein from lutein-enriched eggs is more bioavailable than lutein from supplements or spinach [23]. Long-term lutein intake (15 weeks) also significantly increases the accumulation of lutein in tissues other than serum, such as buccal mucosa cells, adipose, and macula [24].

Both free and esterified forms of lutein are found in nature, though the majority of lutein in foods is in the free form [25]. Lutein esters are more stable and are often used in supplements. Free and esterified lutein are both bioavailable, but supplements containing free lutein may increase the serum/plasma lutein response more than supplements containing lutein esters [26]. Only free lutein is absorbed, so esterified lutein requires an additional ester-hydrolysis step in the small intestine; this could explain the different response between forms [26].

Many host-specific metabolic factors have also been studied, such as single-nucleotide polymorphisms (SNPs) in proteins important for intestinal absorption, transport, and metabolism of lutein. Variants in intestine-specific homeobox (ISX) and microsomal TG transfer protein (MTTP) are associated with differences in postprandial chylomicron lutein response in men [27, 28]. SNPs in ATP-binding cassette (ABC) transporters involved in cellular cholesterol efflux are associated with differences in serum/plasma lutein and zeaxanthin responses and postprandial chylomicron lutein response [28–30]. Some scavenger receptor class B type 1 (SCARB1) and CD36 SNP genotypes are associated with lower circulating levels of lutein [27, 30–32]. Several SNPs in SCARB1 and ABC transporters are associated with lower MP density in women [33].

Serum/plasma lutein concentrations and MP densities are influenced by variables related to both the source of lutein and the host. Our knowledge in this area, particularly related to host factors, is still evolving. With more research, we will be able to make more informed predictions about an individual’s response to dietary sources of lutein.

### Clinical trials on efficacy and dose-response

#### Criterion rationale

These trials help set the efficacious intake level for the bioactive.

#### How Lutein meets this criterion

In clinical trials, lutein/zeaxanthin supplements consistently increase serum lutein/zeaxanthin concentrations and MP density [20]. In one trial, serum lutein concentrations increased linearly as lutein doses increased up to 20 mg/day; similar to other studies, serum concentrations plateaued after 2–3 weeks [34]. MP also followed a linear trend as doses of lutein increased [34]. Supplementing with macular carotenoids (including lutein) slows AMD progression in people with low lutein/zeaxanthin status [35] and improves measurements of visual performance, such as contrast sensitivity, visual acuity, and glare disability [36]. For reducing the risk of AMD, the efficacious intake level for lutein may be ~ 6 mg/day [37].

### Safety data

#### Criterion rationale

Safety data are necessary, even if the bioactive is generally recognized as safe (GRAS).

#### How Lutein meets this criterion

American adults typically consume ~ 1–2 mg lutein/day [4]. A systematic risk assessment of lutein supplements used in placebo-controlled intervention trials was published in 2006 [38]. Based on this assessment, there is strong evidence that lutein is safe up to 20 mg/day [38]. Doses of lutein ranged from 8 to 40 mg/day and study durations have ranged from 7 days to 24 months. Only a few of the studies monitored possible adverse side effects, primarily through self-reporting. Subsequent to this publication, the AREDS2 trial reported no adverse effects—with the exception of some skin yellowing—from lutein and zeaxanthin supplementation (10 and 2 mg/day, respectively) over an average of 5 years in patients with intermediate AMD [35]. In a more recent case study, bilateral “foveal sparkles” were reported in an older woman who took a 20 mg/day lutein supplement for 8 years, along with exceptionally high dietary lutein intake [39]. Seven months after discontinuing the lutein supplement but continuing her dietary habits, the crystals resolved in the right eye, but not in the left eye.

Although safety data are available for lutein, future studies should evaluate chronic consumption of high lutein-containing supplements in specific populations.

### Systematic reviews and meta-analyses

#### Criterion rationale

Systematic reviews and meta-analyses are needed to show efficacy of the bioactive.

#### How Lutein meets this criterion

Based on meta-analyses, there is a significant inverse association between lutein/zeaxanthin intake and risk of late AMD [40] and cataracts [41, 42]. Ma et al. reported that each 300 µg/day increment of dietary lutein/zeaxanthin reduces the risk of nuclear cataracts and posterior subcapsular cataracts by 3% [42]. Lutein/zeaxanthin supplementation is also significantly and dose-dependently related to improvements in visual acuity and contrast sensitivity [43]. Each 1 mg/day increase in lutein/zeaxanthin is associated with a favorable 0.003 reduction on a visual acuity scale (logarithm of minimum angle of resolution, logMAR), as well as improvements in contrast sensitivity of 0.010, 0.007, and 0.006 at the spatial frequencies of 3, 6, and 12 cycles/degree, respectively [43].

### Plausible biological rationale

#### Criterion rationale

Although Lupton et al. [8] state that a biological explanation for efficacy is not required, this criterion is valuable to scientists and evaluators.

#### How Lutein meets this criterion

A lutein-binding protein (steroidogenic acute regulatory domain 3, StARD3) is located in the human macula [44]. MP, which increases in density with increased dietary lutein/zeaxanthin intake, may provide foveal photoprotection against damaging blue light [45]. In addition, lutein/zeaxanthin supplementation modulates the cell density profile of the foveal retinal pigment epithelium (RPE) [46]. This implicates MP in the development and/or maintenance of the RPE, which could thereby reduce susceptibility to macular degeneration.

## Conclusions, challenges, and future directions

Lutein meets all nine criteria proposed by Lupton et al. [8] and should be considered for intake recommendations. Several criteria, namely those related to interindividual metabolic variables and safety data, may require additional attention to strengthen the case for lutein. As mentioned, lutein and zeaxanthin are often found together in foods/supplements and are sometimes jointly reported in food databases and clinical trials. MP optical density also reflects combined lutein/zeaxanthin/*meso*-zeaxanthin content, since all are present in the macula. These points are important to recognize as we consider setting intake recommendations for lutein.

The body of literature linking lutein to a reduced risk of AMD is richer than for the link between lutein and enhanced visual performance. However, using visual performance as the endpoint for intake recommendations would better align with the current dietary guidance for nutrients, which targets healthy populations.

Establishing intake recommendations for lutein would provide the public with yet another reason to eat more of the colorful fruits and vegetables lacking in our diets. Many consumers purchase products/supplements containing lutein, but without a recommendation, they may not be aware of the science that supports its role in health or know the appropriate intake level. The integration of specific intake recommendations for lutein (and potentially other bioactives) into nutrition public policy would also allow for easier evaluation of lutein intakes across populations; this would help assess whether populations are meeting recommendations [8].

Lupton et al. [8] made great strides by proposing an evaluative framework for bioactives, and we have provided supporting arguments that lutein is ready to be considered for DRI-like recommendations. As a next step, key policy makers (NIH, FDA, and others) should convene and discuss the strength of the supporting research, gaps in knowledge, and existing barriers and ways to overcome them. Establishing intake recommendations for lutein will be challenging, but doing so is critically important to Americans who are concerned about optimizing visual performance and reducing their risk of AMD.
